# Intravitreal Dexamethasone Implant for the Management of Choroidal Neovascular Membrane in Pregnancy

**DOI:** 10.7759/cureus.58458

**Published:** 2024-04-17

**Authors:** Imran Mehdi

**Affiliations:** 1 Ophthalmology, Tata Main Hospital, Jamshedpur, IND

**Keywords:** pregnancy, anti-vegf treatment, laser photocoagulation, idiopathic choroidal neovascularization, dexamethasone implant

## Abstract

The management of choroidal neovascularization (CNVM) in pregnant young females has been a lacuna due to the rarity of the condition as well as the non-availability of comprehensive data to showcase the efficacy of currently available treatment regimes in order to achieve a positive outcome for both the growing fetus as well the patient herself. In a review of available literature, the condition has been treated with anti-vascular endothelial growth factors (anti-VEGF), laser photocoagulation, and intravitreal dexamethasone implants (IDI), with varied results ranging from the successful outcome in terms of pregnancy to abortions. When faced with such circumstances, healthcare professionals usually proceed cautiously, balancing the possible advantages against the hazards to the mother and the fetus.

Here we present a case report of a young 30-year-old pregnant lady who developed idiopathic CNVM during her third month of gestation. Being a rare entity, CNVM in young pregnant women raises severe concerns due to potential consequences on the mother's and fetus's health. In certain previously documented cases, pregnant ladies with CNVM have been successfully treated with IDI. Hence, after much deliberation, we chose to go with IDI rather than anti-VEGF, which resulted in the successful management of her CNVM as well as achieving full-term normal delivery without any fetal anomalies. In this particular case, the pregnancy and the visual rehabilitation have both had favorable outcomes. There was no associated increased intraocular pressure (IOP) or changes to the lenticular structure. The literature review also suggests that IDI may still be as effective in managing CNVM during pregnancy, but at a lower risk than anti-VEGF drug. Even with the favorable outcomes revealed in case reports, larger-scale studies to properly examine IDI's safety profile would be required for regulatory clearance of its safety in pregnancy.

## Introduction

Choroidal neovascularization membrane (CNVM) is defined as the growth of new blood vessels that breach through the Bruch's membrane resulting in varied degrees of decrease or blurring of vision. Age-related macular degeneration is the most common predisposing cause for CNVM, but idiopathic CNVM cases have been noted in young individuals without any underlying cause, thus further complicating the clinical process, especially in pregnancy [1]. CNVM can lead to a decline in vision due to retinal deformation caused by fluid accumulation in subretinal spaces [2].

Pregnancy in itself brings about a cascade of changes in normal body physiology, resulting in varying hemodynamic parameters, hormonal fluctuations, and a marked spike in angiogenic growth factors [3]. These events may be attributed to changes in the retinal pigment epithelium and the physiology of the choriocapillaris, thereby exponentially increasing the risk of CNVM development.

In the literature review, CNVM and pregnancy as a coexisting condition have been reported as a complication of presumed ocular histoplasmosis syndrome [4] or punctuate inner chorioretinopathy. In previously documented cases, anti-vascular endothelial growth factors (anti-VEGF) like bevacizumab have been used for the treatment of idiopathic CNVM in limited case series [5,6]. However, their use has also resulted in the termination of pregnancy in a few cases.

Intravitreal dexamethasone implants (IDI) have also been used in the management of cystoid macular edema (CME) secondary to central retinal vein occlusion (CRVO) and other retinal conditions. They like other corticosteroids have the potential to cross the placenta and may affect fetal development. Animal studies have shown adverse effects on fetal development with systemic corticosteroid use. However, there is limited data on the effects of intravitreal administration during pregnancy. Pregnant women with CNVM may experience worsening of vision due to the progression of the condition. Balancing the need for treatment to preserve vision with the potential risks of therapy is crucial. However, the efficacy of these treatments may vary depending on the underlying cause of CNVM. Management decisions for pregnant women with CNVM should involve a multidisciplinary team, including obstetricians, ophthalmologists, and potentially neonatologists, to carefully weigh the risks and benefits of treatment options. Treatment decisions should be individualized based on factors, such as the severity of the CNVM, the gestational age of the fetus, the overall health of the mother, and her preferences. It's essential for pregnant women with CNVM to have open and thorough discussions with their healthcare providers to understand the potential risks and benefits of treatment options and make informed decisions based on their specific circumstances.

## Case presentation

**Figure 1:**
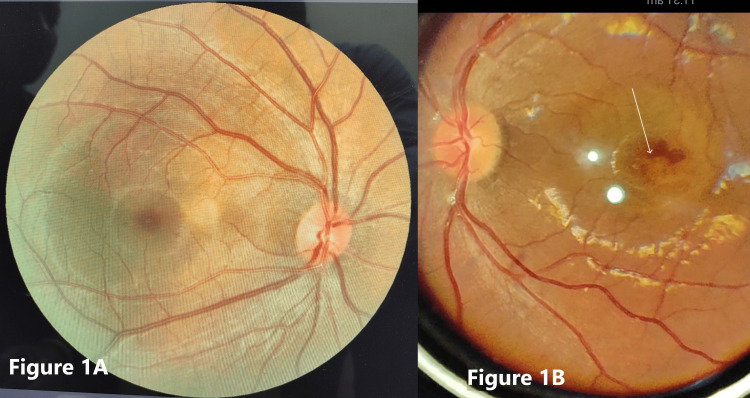
(A) Normal fundus findings in the right eye; (B) Parafoveal sub-retinal hemorrhage in the left eye as marked by a white arrow

A 30-year-old pregnant lady with three months of gestation presented to our eye department with complaints of progressive blurring and a decrease of vision in the left eye for the past one week. The patient had neither proteinuria nor hypertension, and pre-eclampsia was excluded. Her visual assessment revealed a vision of 20/20 OD and 20/40 OS. On refraction, there was no further improvement in her vision in the left eye. Her past ophthalmic history was unremarkable. She underwent a comprehensive ophthalmic examination wherein her intraocular pressure was 12 mmHg in both eyes. Fundus examination revealed a parafoveal sub-retinal hemorrhage in the left eye with a dull foveal reflex (Figures 1A-1B).

**Figure 2:**
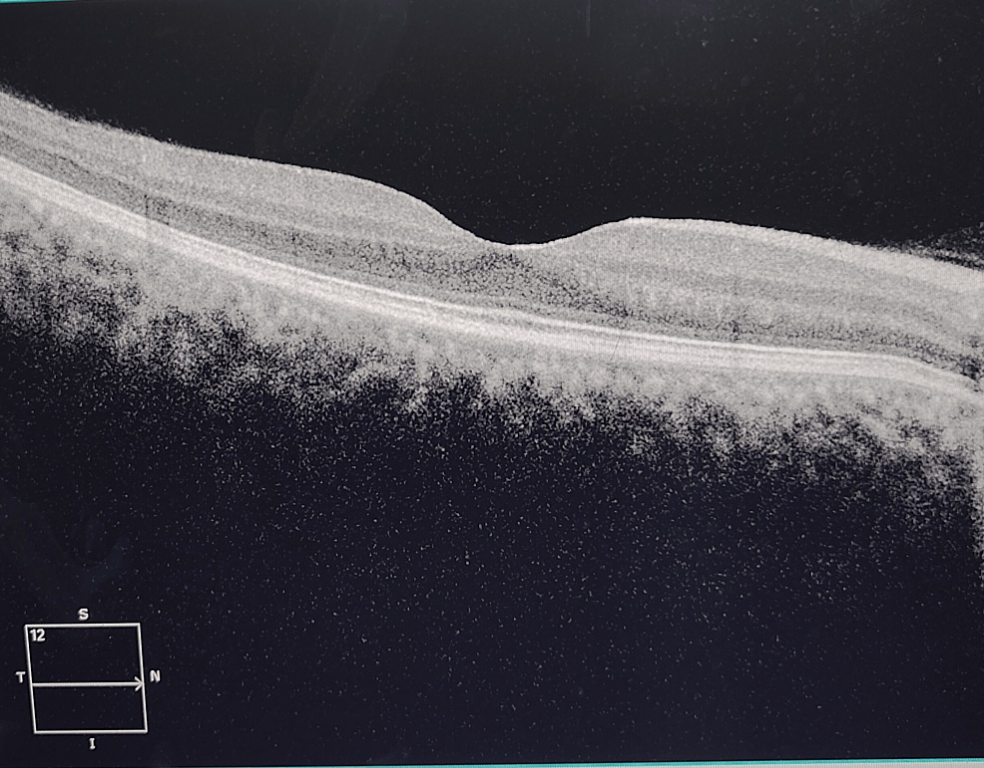
Normal OCT findings in the right eye OCT: Optical coherence tomography

With this clinical picture, a provisional diagnosis of CNVM was proposed, and to further confirm the finding optical coherence tomography (OCT) was done. The OCT findings of the right eye were normal (Figure 2).

**Figure 3:**
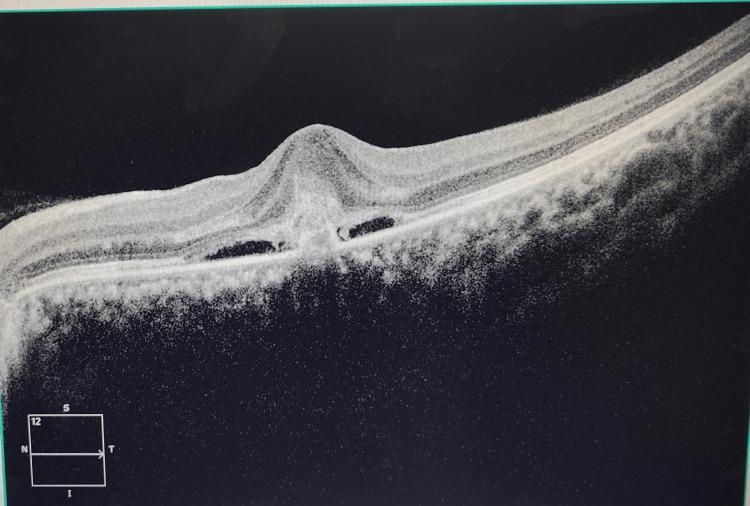
Loss of foveolar contour with subretinal fluid in the left eye

The OCT findings in the left eye revealed a loss of normal foveolar contour with disruption at the inner retinal layers demarcated by an elevated hyper-reflective zone with adjacent hypo-reflective areas at the subretinal space compatible with subretinal fluid and superiorly with fussiness that match with subretinal hyper-reflective material (SHRM) that translates into an active CNVM (Figure 3).

**Figure 4:**
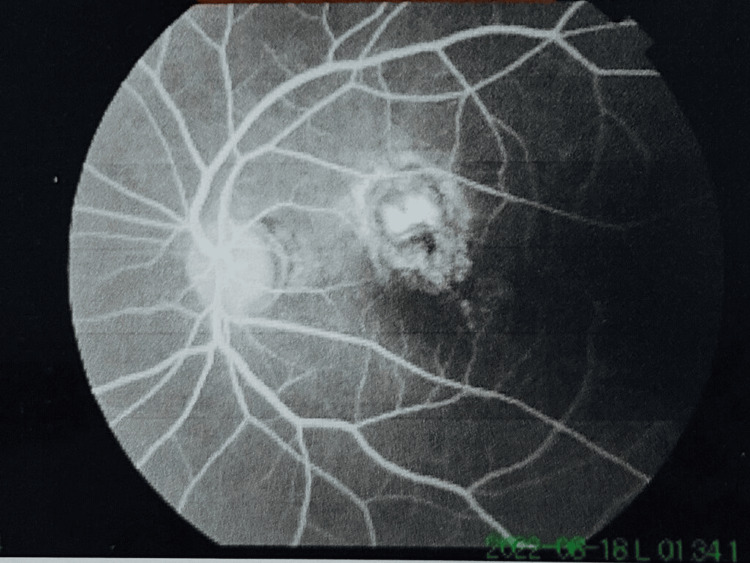
FFA showing late leakage of dye in the left eye around the parafoveal area FFA: Fundus fluorescein angiography

After written consent from the patient, fundus fluorescein angiography (FFA) was carried out to further substantiate the diagnosis. FFA findings showed subfoveal late leakage of dye, thus confirming the diagnosis of CNVM (Figure 4).

FFA and OCT scans showed a juxtapupillary CNVM in the left eye OS. All treatment options were discussed with the patient, of which IDI treatment was accepted by the patient. The post-intravitreal injection period was uneventful. The patient reported for reassessment after one week following injection and then subsequently after one month. The fetal well-being was intensively monitored in the obstetrics department in the intervening period.

**Figure 5:**
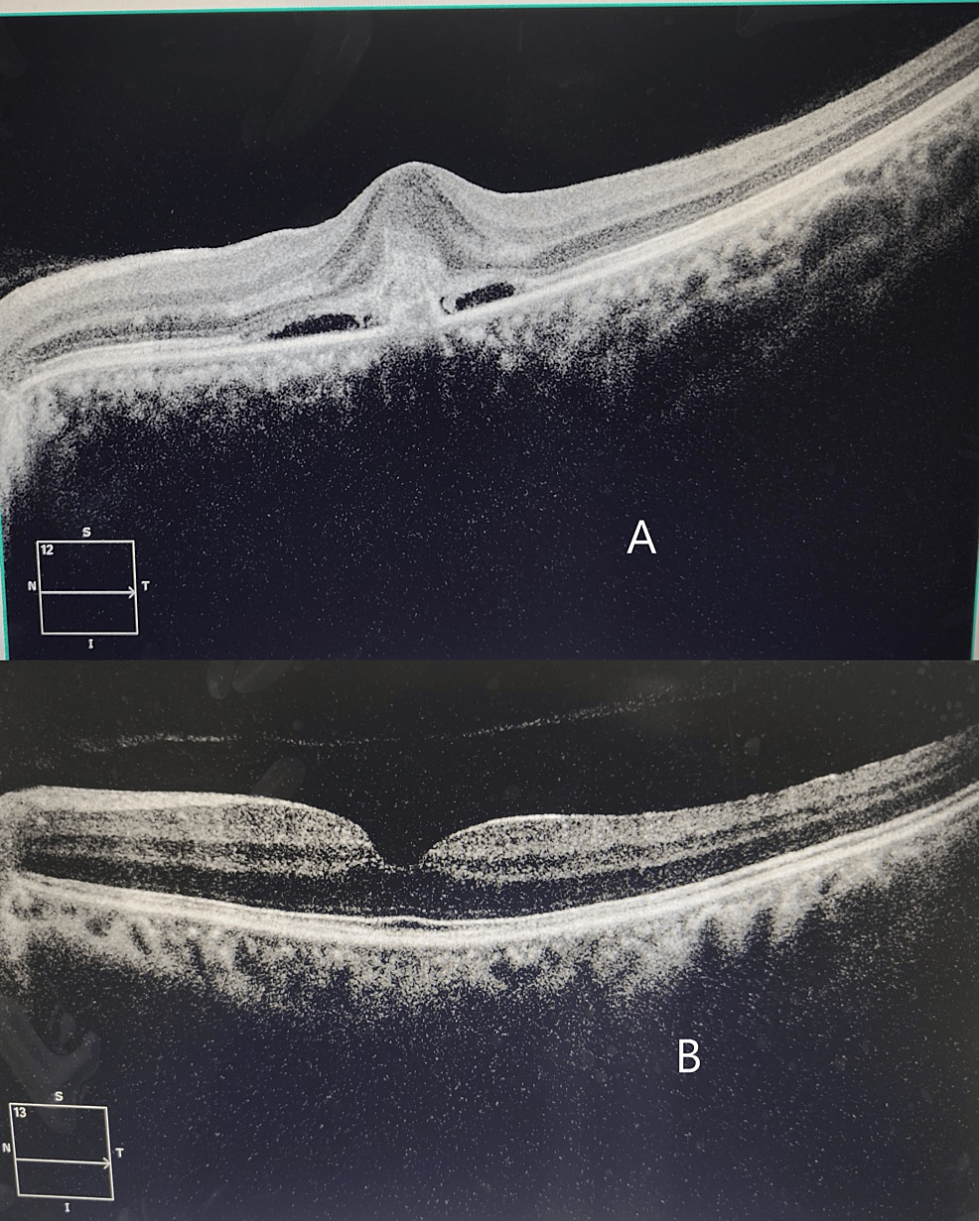
(A) Pre-intravitreal dexamethasone implant OCT in the left eye; (B) Restoration of foveal anatomy and resolution of subretinal fluid post-intravitreal dexamethasone injection OCT: Optical coherence tomography

On her visit after one month, her visual acuity had markedly improved to 20/20 in the left eye. Repeat OCT revealed the absence of subretinal fluid and the tendency towards restoration of normal foveal anatomical architecture (Figures 5A-5B).

She was kept under observation with regular ocular assessment throughout her period of pregnancy. No rise in intraocular pressure or lenticular changes was noted during this period. After six months, she had a normal full-term delivery with no fetal anomalies and no recurrence of ocular symptoms.

## Discussion

Managing CNVM during pregnancy poses notable problems because of the possible dangers to the growing fetus and the mother. Treatment choices are made more difficult by the fact that fetal angiogenesis peaks in the first trimester. There are few clear methods for handling CNVM during pregnancy because of ethical concerns and possible hazards. Medical professionals frequently use a case-by-case approach, carefully balancing the advantages and disadvantages of potential treatments.

The therapy of CNVM often involves the use of intravitreal anti-VEGF medications including ranibizumab, aflibercept, and brolucizumab. Nevertheless, the Food and Drug Administration (FDA) has categorized these pharmaceuticals as category C drugs, meaning that while research on animal reproduction has demonstrated harmful effects on the fetus, sufficient and well-controlled human trials are lacking. Therefore, unless the possible advantages outweigh the hazards, their usage during pregnancy is usually avoided. Although there is little information on the use of anti-VEGF therapies during pregnancy, several studies have linked exposure to these drugs to unfavorable pregnancy outcomes, such as fetal abnormalities, stillbirths, and abortions [7-9]. There have been reported use of anti-VEGF agents for treating CNVM and diabetic macular edema (DME) inadvertently in pregnant female patients, but long-term data is not available [10,11]. Another CNVM treatment option is photodynamic therapy (PDT) with verteporfin. Although it could provide a non-systemic option, there is reason for concern as its safety profile during pregnancy has not been well investigated.

The FDA has authorized IDI for the treatment of DME and CME after CRVO or branch retinal vein occlusion (BRVO). Although the FDA has not authorized IDI, particularly for use during pregnancy, there are reports of it being used off-label in the second and third trimesters to treat disorders including DME and CNVM. The six-month duration of action of IDI might be helpful in minimizing the frequency of injections required during pregnancy. IDI does not specifically target vascular endothelial growth factors (VEGF), in contrast to anti-VEGF therapies. This may be important to pregnant women because of worries about fetal exposure to anti-VEGF drugs. This synthetic glucocorticoid has been used to speed up the development of fetal lungs and is occasionally given clinically to induce preterm labor for this reason [12]. Prenatal drugs such as dexamethasone have also been linked to better baby survival and a lower chance of brain damage in premature babies. In the end, choices on treatment during pregnancy should be made jointly by the patient and their healthcare provider, taking into account the patient's values and preferences in addition to the risks and advantages of the various treatment alternatives.

Given these factors, IDI may be taken into account as a possible course of treatment for CNVM or DME in pregnant individuals, especially where the advantages of treatment exceed the possible dangers. In these circumstances, it is crucial to closely monitor and continuously analyze the health of the mother and fetus in order to guarantee the best possible outcomes for all parties.

## Conclusions

Although CNVM in young pregnant women is uncommon, the possible effects on the health of the mother and fetus make it a serious concern. Pregnant individuals with CNVM have been effectively managed with IDI in recorded situations. Both the results of the pregnancy and the visual rehabilitation in these circumstances have been positive. The use of IDI during pregnancy has not been linked in any of these cases to elevated intraocular pressure (IOP) or lenticular alterations. IDI may be less harmful than anti-VEGF medications when pregnant and still be just as effective at managing CNVM. Larger-scale studies to fully evaluate IDI's safety profile would be necessary for formal approval of its safety in pregnancy, even with the encouraging results shown in case reports.

In conclusion, as per the result of our study, there is encouraging data in favor of IDI usage in pregnant women with CNVM, though more investigations including large-scale studies are required to conclusively determine the treatment's safety and effectiveness in this group. In order to effectively manage CNVM during pregnancy, individualized decision-making that weighs the advantages and disadvantages of treatment alternatives is still essential. To guarantee the best outcomes for the mother and the fetus, close cooperation between obstetricians and ophthalmologists is necessary.
